# SARS-CoV-2 integral membrane proteins shape the serological responses of patients with COVID-19

**DOI:** 10.1016/j.isci.2021.103185

**Published:** 2021-09-29

**Authors:** Sophie Martin, Christopher Heslan, Gwénaële Jégou, Leif A. Eriksson, Matthieu Le Gallo, Vincent Thibault, Eric Chevet, Florence Godey, Tony Avril

**Affiliations:** 1 Inserm U1242 Oncogenesis Stress Signaling, 35000 Rennes, France; 2 Centre Eugène Marquis, 35000 Rennes, France; 3 Department of Virology, CHU Pontchaillou, 35000 Rennes, France; 4 Department of Chemistry & Molecular Biology, University of Gothenburg, 41390 Göteborg, Sweden

**Keywords:** Immunology, Virology

## Abstract

Severe acute respiratory syndrome coronavirus 2 (SARS-CoV-2) pandemic has elicited a unique mobilization of the scientific community to develop efficient tools to understand and combat the infection. Like other *coronavirae*, SARS-CoV-2 hijacks host cell secretory machinery to produce viral proteins that compose the nascent virions; including spike (S), envelope (E), and membrane (M) proteins, the most exposed transmembrane proteins to the host immune system. As antibody response is part of the anti-viral immune arsenal, we investigate the immunogenic potential of S, E, and M using a human cell-based system to mimic membrane insertion and N-glycosylation. Both S and M elicit specific Ig production in patients with SARS-CoV-2. Patients with moderate and severe diseases exhibit elevated Ig responses. Finally, reduced Ig binding was observed with spike G614 compared to D614 variant. Altogether, our assay points toward an unexpected immune response against M and represents a powerful tool to test humoral responses against actively evolving SARS-CoV-2 variants and vaccine effectiveness.

## Highlights

• SARS-CoV-2 membrane proteins antigenicity was evaluated in a human cell-based assay
• The assay recapitulates their membrane insertion and post-translational modifications
• High Ig response against S and M proteins was observed in severe patients with COVID-19
• The D614 variant of spike exhibited an altered structure and reduced Ig binding

## Graphical abstract

**Figure:**
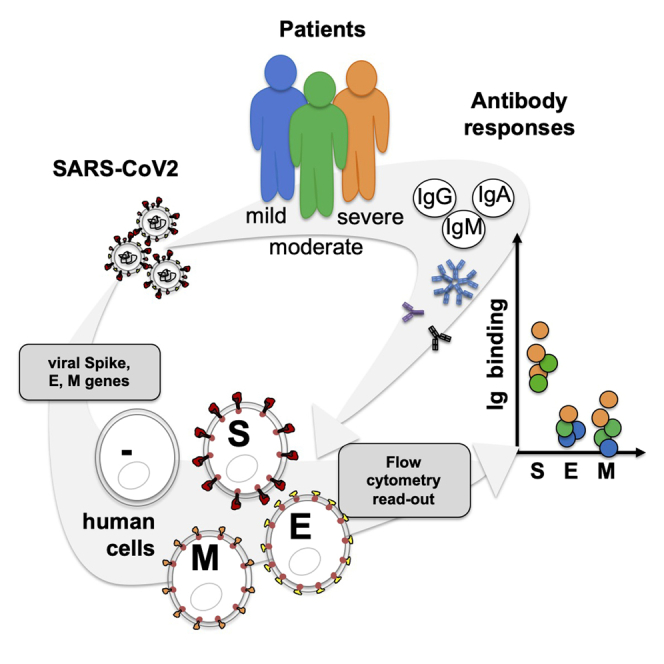

## Introduction

The current coronavirus disease 2019 (COVID-19) pandemic has triggered unprecedented collective research efforts from the scientific community to better understand the disease and its cellular and molecular mechanisms to identify efficient therapeutic drugs for taking care of infected patients with the severe acute respiratory syndrome coronavirus 2 (SARS-CoV-2) and to develop vaccines for protecting the whole population from the infection (Hu et al., 2020; Poland et al., 2020; Sicari et al., 2020). One of the initial challenges in the fight against this virus was to rapidly detect SARS-CoV-2-infected patients to limit the propagation of the virus through isolation (Ravi et al., 2020). Another challenge was to better understand the global antibody responses against SARS-CoV-2 proteins in patients (Poland et al., 2020; Zohar and Alter, 2020).

Among the anti-viral immune responses elicited in infected patients, immunoglobulin (Ig) responses against viral transmembrane proteins expressed at the surface of the virus envelope are important for generating antibodies that limit virus propagation. This occurs by preventing interactions with host cells, *i.e.*, production of neutralizing antibodies that block the binding of the viral transmembrane spike protein to its receptor angiotensin-converting enzyme 2 (ACE2) expressed by infected host cells (Poland et al., 2020; Lan et al., 2020; Hoffmann et al., 2020; Zohar and Alter, 2020). These anti-virus antibodies are also key mediators to trigger antibody-dependent immune responses such as the complement-dependent cytotoxicity as part of the humoral response (Zohar and Alter, 2020) or the antibody-dependent cellular cytotoxicity mediated by immune cells harboring Fc receptors such as NK lymphocytes, macrophages, and granulocytes to allow phagocytosis and destruction of the virus (Zohar and Alter, 2020).

Some of these cellular actors such as macrophages and neutrophils could also contribute to the aggravation of the disease by releasing chemokines and cytokines that enhance inflammatory cascades described as “cytokine storms” leading to lesions of infected tissues; although, the involvement of the antibody-dependent mechanisms still need to be confirmed in patients with COVID-19 (Zohar and Alter, 2020). Most of the serological assays developed against SARS-CoV-2 are based on the recognition of the viral transmembrane spike protein and the nucleocapsid protein N, considered as major targets of antibody responses (Grzelak et al., 2020; Ripperger et al., 2020). Besides the viral spike protein, little is known on Ig responses toward the others viral transmembrane proteins E and M also directly exposed to the host immune system.

Spike (S), envelope (E), and membrane (M) are integral membrane proteins that transit through the host cells’ endoplasmic reticulum (ER), the ER-Golgi intermediate compartment (ERGIC), and most likely the Golgi complex. In the ER, these proteins are N-glycosylated, folded, and assembled in the ERGIC for virus budding and release (Sicari et al., 2020). This maturation process is key for proper viral protein functions. For instance, spike N-linked glycosylation is required for virus entry into the host cells impacting directly on spike stability during its synthesis instead of its binding ability to the ACE2 receptor (Yang et al., 2020). These modifications might be also key for antibody recognition.

In the present study, we relied on an experimental system that recapitulates protein modifications acquired through the host cells’ secretory pathway to explore the antibody responses of SARS-CoV-2-infected patients. We found that S and M proteins (but not E) exhibited antigenic domains recognized by IgG, IgM, and IgA in SARS-CoV-2-infected patients. High levels of Ig responses were observed in patients with COVID-19 with moderate and severe forms of the disease. Finally, SARS-CoV-2 spike D614 and G614 variants were compared, showing reduced Ig binding on the spike G614 variant. Altogether, this study underlines the necessity of considering the mammalian cellular system to better characterize the serological status of patients with COVID-19.

## Results

### Expression of spike, E, and M in mammalian cells and antibody-based detection of mature integral membrane proteins

**Figure 1:**
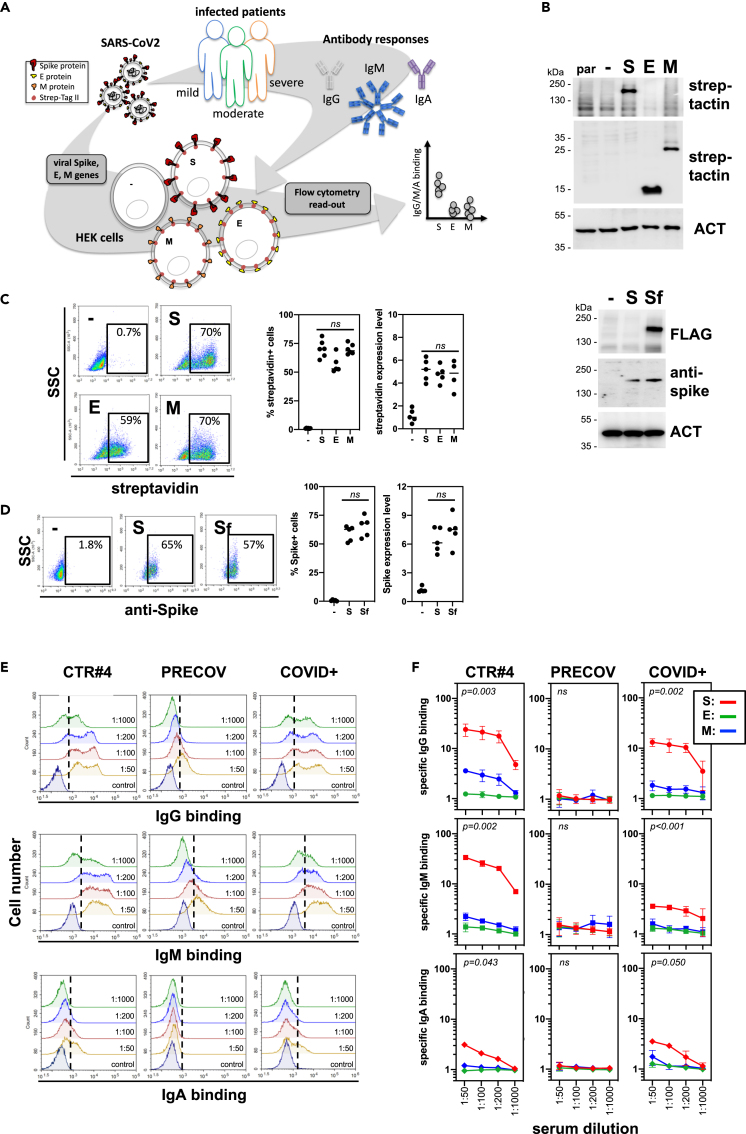
Development of a serological assay that mimics surface expression of SARS-CoV-2 integral membrane proteins (A) Strategy and workflow of the SARS-CoV-2 serological assay developed using flow cytometry. HEK cells transiently transfected with viral genes encoding integral transmembrane proteins (*i.e.* E, M, and spike) were used as matrix for the detection of antibodies present in sera obtained from patients with COVID-19. The binding of anti-viral transmembrane protein antibodies of IgG, IgM, and IgA subtypes was analyzed by flow cytometry. Results were expressed as specific antibody binding to viral transmembrane proteins as non-specific binding was determined using non-transfected HEK cells. (B–D) Expression of viral integral membrane proteins at the surface of HEK cells. HEK cells transfected with viral genes encoding E, M, and spike transmembrane proteins tagged with two Strep Tag motifs were analyzed for viral protein expression by western blot (B) and flow cytometry (C and D) using StrepTactin, streptavidin, or anti-Spike antibody. Percentage of positive cells and surface expression levels was represented in (C) and (D). Statistical analysis: paired two-tailed *t test* comparing S versus E, S versus M and E versus M conditions (C); and S versus Sf condition (D). (E and F) Detection of immunoglobulin IgG, IgM, and IgA binding at the surface of HEK cells expressing viral transmembrane proteins by flow cytometry. Positive sera from SARS-CoV-2-infected patients (CTR#4 and COVID+) were incubated with HEK cells expressing E, M, and spike viral proteins at different dilutions. Serum from a healthy donor (PRECOV) obtained before January 2020 was used as a negative control. The binding of IgG, IgM, and IgA immunoglobulins was analyzed by flow cytometry using secondary antibodies specific for each Ig subtype. Representative flow cytometry histograms were shown for spike in (E), and results were presented as specific Ig binding relative to HEK cells (F). Statistical analysis: one-way ANOVA test comparing S, E, and M conditions.

As viral transmembrane protein recognition is part of the anti-SARS-CoV-2 immune response, we developed a mammalian cell-based serological assay using SARS-CoV-2 S, E, and M expressing human embryonic kidney (HEK) cells to mimic integral membrane protein maturation found at the surface of viral particles (Figure 1A). HEK cells were transiently transfected with genes encoding for SARS-CoV-2 S, E, and M proteins in tandem with either two Strep-Tag II motifs (Gordon et al., 2020) or an FLAG tag (hereafter named Sf). Forty-eight hours after transfection, total and surface expression of S, E, and M was confirmed using both western blotting with HRP-conjugated StrepTactin (Figure 1B), anti-FLAG or anti-Spike antibodies, and flow cytometry using BV650-conjugated streptavidin, respectively (Figure 1C). Cell surface expression of S was also confirmed using flow cytometry using an anti-spike antibody (Figure 1D). The proportion of positive cells and the expression levels of viral proteins were similar between experiments and when viral proteins were compared (Figures 1C and 1D). To validate the binding of anti-SARS-CoV-2 IgG, IgM, and IgA subtypes to HEK cells expressing viral transmembrane proteins, we used two sera from SARS-CoV-2-infected patients (COVID+ and CTR#4, the latter being distributed by SeroBio as a validation tool for diagnostic laboratories) and one serum from a healthy donor (PRECOV obtained before January 2020) as a negative control. Sera were incubated with non-permeabilized HEK cells, and Ig binding was detected using secondary antibodies specific for each Ig subtype. Non-specific binding was determined using non-transfected HEK cells. IgG, M, and A binding was observed on S-expressing HEK cells using sera form SARS-CoV-2-infected patients in a concentration-dependent manner, whereas no Ig binding was detected using the healthy serum (Figures 1E and 1F). These results indicate that S is expressed at the surface of HEK cells and can be detected by anti-S antibodies from COVID+ patients.

### Anti-S and M serological responses in COVID+ patients

**Figure 2:**
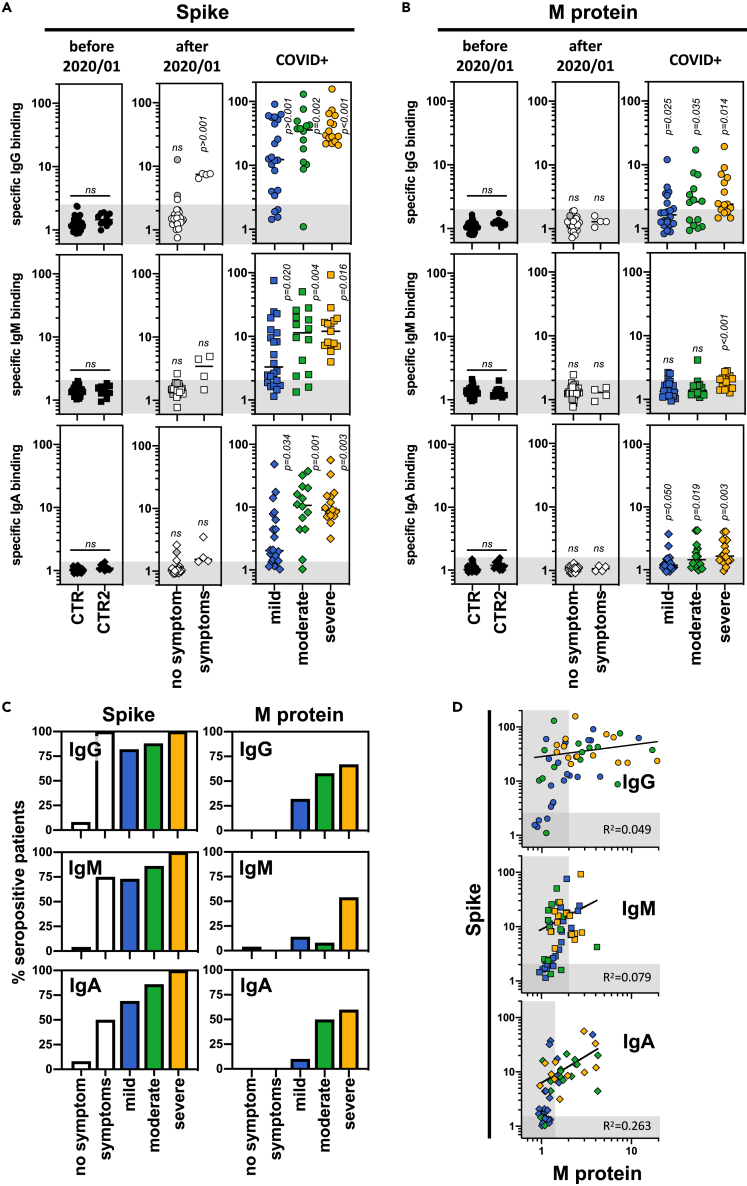
Serological profile of SARS-CoV-2-infected patients against viral integral membrane proteins according to the disease severity (A and B) Sera from control donors and SARS-CoV-2-infected patients were tested for their positivity against viral spike (A) and M proteins (B) using the SARS-CoV-2 serological assay described in Figure 1. Control sera were obtained from healthy donors and collected before January 2020 (CTR, n = 38) and from patients infected with other coronaviruses (n = 5) or patients with hyperimmunoglobulin M syndrome (n = 5) (included in CTR2, n = 10). Sera collected after January 2020 were obtained from donors without symptoms (no symptom, n = 26); with symptoms related to SARS-CoV-2 infection (symptoms, n = 4); and from patients positive for SARS-CoV-2 infection (COVID+) and developing mild (blue, n = 22), moderate (green, n = 14), and severe (orange, n = 15) forms of COVID-19. Specific binding of IgG (circles), IgM (squares), and IgA (diamonds) were represented, and thresholds (gray boxes) were obtained with the basal levels of Ig binding from control sera. Statistical analysis: unpaired two-tailed *t test* with Welch's correction comparing CTR versus CTR2 donors and (CTR + CTR2) versus no symptom, symptoms, mild, moderate, or severe donors. (C) The percentage of seropositive patients from the different groups was calculated using thresholds obtained in (A and B). (D) Correlation between anti-spike and anti-M Ig responses was represented including sera from patient with COVID-19 developing mild (blue, n = 22), moderate (green, n = 14), and severe (orange, n = 15) forms of COVID-19.

A cohort of 129 patients was next tested in our serological assay including sera from (i) heathy/asymptomatic donors obtained before (n = 38) and after January 2020 (n = 26), (ii) patients infected with non-SARS-CoV-2 coronavirus (n = 5), (iii) patients suffering from hyper-immunoglobulin M syndromes (n = 5), (iv) patients with symptoms similar to those observed in COVID+ patients (*i.e.* anosmia, cough, fatigue, fever) (n = 4), and (v) patients previously infected with SARS-CoV-2 (as confirmed by PCR, n = 51) and developing mild (patients who did not need hospitalization, n = 22), moderate (hospitalized patients treated with oxygen therapy <5L, n = 14), and severe (hospitalized patients in ICU with oxygen therapy >5L or intubated, n = 15) forms of the COVID-19 disease (Tables 1 and S1). The time between the PCR tests (or the first symptoms) and the blood sampling was similar between the groups of patients with COVID-19 (between 15 and 25 days; Figure S1A). Our results were comparable to those obtained with assays developed for diagnostic laboratories by Beckman (IgG anti-spike) and Roche (Ig anti-N protein), showing 93% and 89% concordance, respectively (Figure S1B). No Ig binding to S, E, and M proteins was observed using (i) control sera including those obtained before January 2020, (ii) sera from patients infected with other coronaviruses, or (iii) sera from patients suffering from hyper-immunoglobulin M syndromes (Figures 2A, 2B, and S1C). The positive threshold was therefore set using these control sera. Anti-spike IgG, IgM, and IgA were detected in sera from COVID+ patients as well as in those from patients with COVID-19-associated symptoms. Anti-spike Ig titers were higher in patients with moderate and severe forms of the disease compared to mild forms (Figures 2A and 2C). Anti-E Ig titers were never detected in any of the tested sera (Figure S1C). Importantly, anti-M Ig titers were also observed at a higher level in patients with severe forms of the disease (Figures 2B and 2C). Interestingly, while sera positive for anti-M Ig always exhibited anti-spike Ig signals, some anti-spike Ig-positive sera did not show any detectable anti-M Ig (Figure 2D).

**Table 1:** - Clinical features of the cohort used in this study

	Age (mean ± SD)	Sex (% F/M)	Time from PCR	Time from symptoms	Symptoms/treatments
**Before 20/01**					

CTR (n = 38)	37 ± 14.8	54/46	–	–	–
CTR2 (n = 10)	33 ± 28.8	50/50	–	–	–
Coronavirus (n = 5)	45 ± 25.1	25/75	59 ± 39.3	–	–
Hyper-IgM syndrome (n = 5)	22 ± 30.5	75/25	–	–	–

**After 20/01**					

No symptom (n = 26)	38 ± 10.4	62/38	–	–	Two donors contact persons
Symptoms (n = 4)	50 ± 18.9	50/50	130 ± 21.6	–	Cough, fatigue, fever

**COVID+**					

Mild (n = 22)	48 ± 26.8	77/23	41 ± 53.2	52 ± 63.6	Cough, fatigue, fever, anosmia
Moderate (n = 14)	72 ± 16.1	42/58	14 ± 14.7	25 ± 16.8	Lung damage/hospitalization, O_2_ therapy (2.6 + 0.96)
Severe (n = 17)	64 ± 11.2	34/66	15 ± 8.1	25 ± 8.2	Hospitalization, O_2_ therapy (11 ± 6.2), intubated

### Reduced Ig binding to spike G614 compared to D614 in COVID+ patients

**Figure 3:**
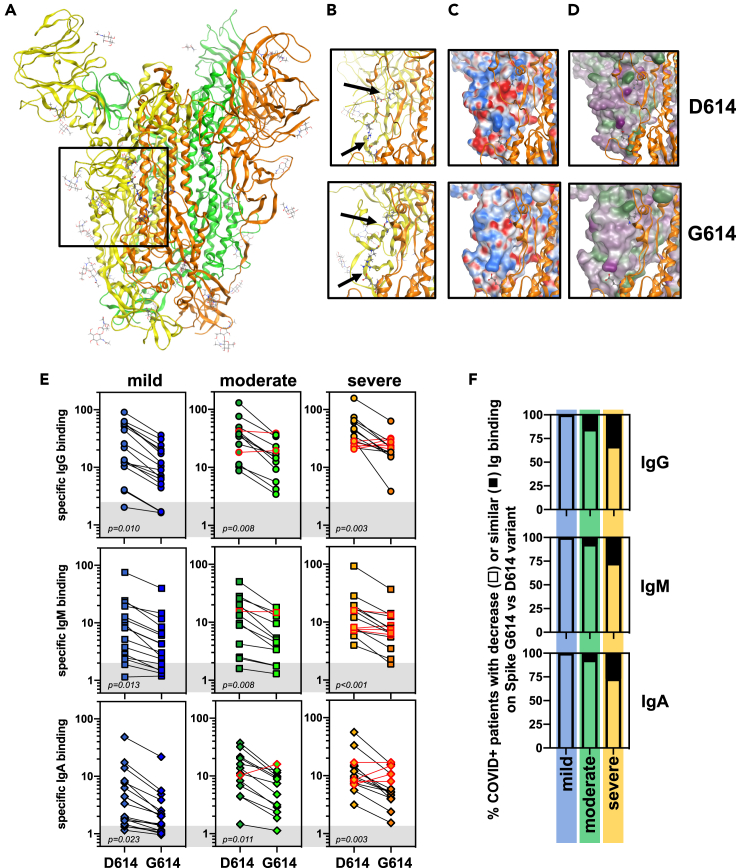
Impact of spike G614 variant on the seropositivity of SARS-CoV-2-infected patients (A–D) Protein structure of spike obtained from the Protein Data Bank (PDB ID6zb5 for D614 variant and PDB ID6xs6 for G614 variant). The region in which the amino acid 614 was localized (square) on the trimer of spike molecules (in green, orange, and yellow) (A) was further analyzed in (B) for spike D614 and G614 variants. Predicted residue interactions with electrostatic (C) and lipophilic (D) properties were compared between spike D614 and G614 variants. (E) Sera from patients with COVID-19 developing mild (blue, n = 17), moderate (green, n = 13), and severe (orange, n = 15) forms of COVID-19 were re-evaluated in the SARS-CoV-2 serological assay using spike D614 and G614 expressing HEK cells. Specific binding of IgG (circles), IgM (squares), and IgA (diamonds) were represented, and thresholds (gray boxes) were obtained with the basal levels of Ig binding from control sera tested in Figure 2A. Statistical analysis: unpaired two-tailed *t test* with Welch's correction comparing D614 versus G614 conditions. (F) Percentage of patients with decreased seropositivity against D614 and G614 variants from the different groups were presented.

Our assay recapitulating viral protein modification and insertion in a membrane was validated using patients' sera with COVID-19 and yielded results comparable to those obtained with commercially available tests (Figure S1B). However, the latter tests use the spike D614 variant as antigen, and it is well established that most European patients until the end of 2020 were mainly infected by SARS-CoV-2 expressing spike G614 (*e.g.* French patients were exclusively exposed to spike G614 variant from March to December 2020 (Korber et al., 2020)). This might lead to biases in data interpretation. Hence, we sought to investigate potential differences in terms of antibody responses using our assay. First, we compared the structural properties of the spike D614 and G614 variants. As a very useful tool to provide overviews of large protein complexes, cryo-electron microscopy has been extensively used to describe SARS-CoV-2 spike structural features (Benton et al., 2021; Zhang et al., 2020; Gobeil et al., 2021). One limitation of such an approach is the low resolution in terms of atomic details. We performed a molecular modeling focusing on the beta-sheet-rich domain containing D/G614 of chain A (yellow), and its interaction with the patch T824-E865 on chain B (golden), squared in Figure 3A. D614 (chain A) forms an inter-protomeric salt bridge to K854 (chain B) (Figure 3B top). In the same region, there is also an inter-protomeric salt bridge between R646 (chain A) and E865 (chain B). Using the model with the D614G mutant of 6BZ5, we observed that K854 (chain B) remains pointing toward protomer A and forms an H-bond to the carbonyl backbone of G614 (*i.e.* a weaker and more strained interaction than in the case of D614 (Figure 3B, bottom)). The second salt bridge R646 (chain A) → E865 (chain B) is still retained. Looking at the electrostatic and hydrophobic properties of the area in protomer A, we observed that the domain side interacting with protomer B is largely nonpolar, except for D614 and R646 (Figures 3C and 3D, top). In contrast, the electrostatic interaction is considerably weaker in the G614 variant and essentially only retained by R646 protruding toward E854 (chain B) (Figures 3C and 3D, bottom). The sequences at the domain interfaces consist largely of non-polar residues, and the change from D614 to G614 clearly impacts on the overall polarity of the protomer A interaction area. We also note that in the G614 variant, the loop region after K854 of protomer B (golden) is bending further away from protomer A, than what is observed in the D614 variant. Analysis of the surfaces of protomer B in the interface also illustrates the difference in interactions between the two protomers. In particular, the non-polar region of protomer B is protruding toward protomer A between D614 and R646 in the D614 variant but is in contrast pushed back/out in the G614 variant (Figure S2E). In addition, the segment around K854 is in G614 clearly rotated away from protomer A. In the region close to E865 of protomer B, an increased exposure of hydrophilic/polar residues toward the solvent (better seen in the lipophilicity surfaces) was observed. These analyses indicate that the G614D mutation might alter the global structure of the protein and therefore the antigenic response.

Anti-spike Ig response was therefore re-evaluated using sera from COVID+ patients and anti-spike D614 and G614 variants expressing cells. Expression of the spike variants in HEK cells was validated as previously (Figures S2B–S2D). Similar expression levels of spike D614 and G614 variants were found at the cell surface (Figures S2C and S2D). Sera from COVID+ patients were then tested on both cell systems and a lower anti-S IgG, IgM, and IgA binding to spike G614 was observed in most of the patients than that observed for binding to the D614 variant (Figure 3E). Only a small proportion of those patients (less than 15%) displayed similar Ig responses against the two spike variants (Figures 3E and 3F). When compared to assays developed for diagnostic laboratories by Beckman (IgG anti-spike) and Roche (Ig anti-N protein), respectively, 90% and 95% concordance were observed with our cell-based assay using G614 variant (Figure S2E). Hence, our experimental system allows for discrimination between anti-S D614 vs. G614 Ig signals likely due to the advantage of using transmembrane-inserted spike following complex folding and post-translational modifications.

## Discussion

Using a mammalian cell-based assay with the SARS-CoV-2 integral membrane proteins S, E, and M (Figure 1), we identified antibody responses against both S and M proteins in COVID+ patients but not against the transmembrane viral protein E. Higher anti-S and anti-M Ig production correlated with symptom severity in hospitalized COVID+ patients (Figure 2). Furthermore, although French patients were exclusively exposed to SARS-CoV-2 expressing the spike G614 variant until December 2020, reduced IgG, IgM, and IgA binding was observed on spike G614 compared to that observed for the D614 variant (Figure 3). Overall, this study underlines the importance of using antigens respecting viral protein constraints to investigate antibody responses against SARS-CoV-2 transmembrane proteins.

Most of the diagnostic tests currently used to detect the antibody response against SARS-CoV-2 target the viral proteins S and N as both were initially found to be expressed abundantly and exhibit substantial antigenicity (Algaissi et al., 2020; Amanat et al., 2020; Lee et al., 2020; Houlihan and Beale, 2020; Rikhtegaran Tehrani et al., 2020; La Marca et al., 2020). One of the drawbacks associated with these assays is the use of recombinant proteins produced in prokaryotic systems that only include fragments of spike (*e.g.* S1 subunit or RBD domain (Algaissi et al., 2020; Amanat et al., 2020; Lee et al., 2020; Houlihan and Beale, 2020; Rikhtegaran Tehrani et al., 2020; La Marca et al., 2020)). Recent structural analyses have revealed that the structural integrity of the full-length spike multimers (trimers, dimers of trimers, and more) is important to better understand its immunogenic potential (Bangaru et al., 2020). As the virus hijacks the host secretory machinery to produce nascent viral particles in infected cells (Sicari et al., 2020), we designed a reliable serological assay using mammalian cells that express full-length SARS-CoV-2 transmembrane proteins S, M, and E, the most exposed to the host immune system. Such a system allows for proper folding and post-translational modifications of the viral proteins (Sicari et al., 2020). These modifications include, for instance, disulfide bond formation and N-glycosylation, thus leading to native structural features.

Recent studies have mapped the regions of SARS-CoV-2 proteins recognized by antibodies from patients with COVID-19 using proteome microarrays (Krishnamurthy et al., 2020; Wang et al., 2020; Jiang et al., 2020). Antibody production was detected against peptides derived from the structural proteins spike (S1, S2, and RBD domains), N, and M and from the accessory protein ORF3a. Interestingly, we are able to demonstrate for the first time the occurrence of an antibody response in COVID+ patients against the entire M protein using our cell-based serological assay, thereby confirming results observed in proteome microarrays in a more physiological context (Krishnamurthy et al., 2020; Wang et al., 2020; Jiang et al., 2020). Higher Ig binding was observed in hospitalized COVID+ patients with moderate and severe forms of the disease. Of note, Ig response against the M protein was always observed in patients that also exhibited an anti-spike Ig response.

One additional advantage of the assay developed herein is the possibility to quickly adapt to express new envelope protein variants. As an example, we compared the Ig responses against the spike variants D614 and G614. To our surprise, spike G614 displayed a lower Ig binding capacity compared to D614. Structural analyses revealed differences in electrostatic and hydrophobic surfaces between the spike variants that could impact on Ig affinity, as discussed previously (Benton et al., 2021). Interestingly, D614G is located near the 615 to 635 flexible loop leading to a salt bridge between D/G614 of one protomer and K854 on the neighboring protomer, possibly affecting the global structure of the spike trimer (Zhang et al., 2020), increasing the RBD “up” state and S1/S2 proteolysis (Gobeil et al., 2021). Of note, no difference in Ig binding levels has previously been observed in ELISA-based assays (Klumpp-Thomas et al., 2020). The discrepancy observed with our study could be linked to the type of serological assay used; ELISA versus cell-based assays could exacerbate these protein structural differences. Intriguingly, French patients with COVID-19 analyzed in this study were exclusively exposed to SARS-CoV-2 expressing spike G614 variant, suggesting that the mutation does not reduce the antigenicity against spike but instead reduces IgG, IgM, and IgA binding. One could speculate that this mutation gave a selective advantage to the European SARS-CoV-2 strain by limiting the antibody response in infected patients, therefore allowing larger virus spreading. Many new SARS-CoV-2 strains associated with spike substitutions have recently emerged (e.g. as observed for variants from Brazil, United Kingdom, and South Africa) with increased infectivity (Leung et al., 2021; Tegally et al., 2020; Paiva et al., 2020; Gröhs Ferrareze et al., 2021). These new strains also display several mutations in other viral genes encoding for structural and accessory proteins. However, very few mutations were described for M so far (Mercatelli and Giorgi, 2020; Laamarti et al., 2020), suggesting that anti-M Ig responses described in this study could be conserved across the different SARS-CoV-2 lineages. If anti-M antibodies effectively reduce SARS-CoV-2 infectivity, the low variation burden on this protein might also reveal an efficient tool for vaccine development.

### Limitations of the study

In contrast to the other SARS-CoV-2 transmembrane proteins spike and M, no antibody against the protein E was detected in our assay. This could be explained by the fact that E might not be expressed at the surface of the cells or that it may not expose enough antigenic regions to mount a potent immune response. Previous studies indicate that SARS-CoV-2 derived E protein seems to be mainly localized in the host cells at the ER, ERGIC, and/or Golgi compartments, although the precise localization is still debated (Satarker and Nampoothiri, 2020; Miserey-Lenkei et al., 2020). Furthermore, a recent study unveils the impact of the addition of tags on E protein localization within the host cells, in particular at the C-terminus position (near the DLLV motif) leading to its retention into the ER (Pearson et al., 2021). We could not, however, exclude that our construct encoding for a viral E protein tagged with two Strep-Tag motifs may also lead to its retention within the host cells. Another limit of our study lies in the fact that viral S, E, and M proteins were expressed individually and not simultaneously; the latter possibly leading to structural changes and variation in the presentation of antigenic regions. Moreover, the experimental cell model used might also be a source of bias in post-translational modifications and presentation of S, E, and M at the cell surface. At last, a real added-value to our study would be to characterize how anti-M antibodies may impact on SARS-CoV-2 infectivity and could participate to the virus clearance.

At the time of massive vaccination against spike using the RNA-based approaches developed by Pfizer-BioNTech and Moderna (Walsh et al., 2020; Anderson et al., 2020) and the spreading of novel SARS-CoV-2 strains carrying spike mutations, our serological assay represents a reliable test to verify the immunization efficiency during the vaccination and to analyze the impact of spike mutations on antibody responses.

## STAR★Methods

### Key resources table

**Table:** 

REAGENT or RESOURCE	SOURCE	IDENTIFIER
**Antibodies**		

Donkey polyclonal anti-rabbit IgG (H+L) Alexa Fluor 488	Jackson Immunoresearch, Ely, UK	Cat# 711-545-152; RRID: AB_2313584
Donkey polyclonal F(ab’)2 anti-human IgG (H+L) Alexa Fluor 488	Jackson Immunoresearch, Ely, UK	Cat# 709-546-149; RRID: AB_2340569
Donkey polyclonal F(ab’)2 anti-human IgM Fc5μ Alexa Fluor 647	Jackson Immunoresearch, Ely, UK	Cat# 709-606-073; RRID: AB_2340579
Goat polyclonal F(ab’)2 anti-human IgA FITC	Thermo Fisher Scientific, Illkirch, France	Cat# A24459; RRID: AB_2535928
Goat polyclonal anti-rabbit Ig HRP	Dako, Les Ulis, France	Cat# P0448; RRID: AB_2617138
Mouse monoclonal anti-FLAG M2 HRP	Sigma-Aldrich, St Quentin-Fallavier, France	Cat# F1804-1MG; RRID: AB_262044
Rabbit monoclonal anti-SARS-CoV-2 Spike S1	Sino Biologicals, Wayne, PA, USA	Cat# 40150-R007; RRID: AB_2827979

**Bacterial and virus strains**		

XL10-gold ultracompetent cells	Agilent Technologies, Les Ulis, France	200315
Biological samples		
Donkey serum	Sigma-Aldrich St Quentin-Fallavier, France	S30-100ML
Plasmid pCMV3 SARS-CoV-2 Spike (B1.1.7) C-FLAG tag - Hygromycin	Sino Biologicals, Wayne, PA, USA	VG40771-CF
Plasmid pLVX EF1alpha nCoV2019 E IRES-Puro	Krogan laboratory UCSF San Francisco, CA, USA	Gordon et al., 2020 PMID: 32353859
Plasmid pLVX EF1alpha nCoV2019 M IRES-Puro	Krogan laboratory UCSF San Francisco, CA, USA	Gordon et al., 2020 PMID: 32353859
Plasmid pTwist EF1alpha nCoV-2019 S (D614 variant) 2xStrep	Krogan laboratory UCSF San Francisco, CA, USA	Gordon et al., 2020 PMID: 32353859
Serum samples –SEROCOV collection	Rennes Biobank, Rennes BRIF: BB-0033-00056	DC-2019-3585

**Chemicals, peptides, and recombinant proteins**		

7AAD	BD Biosciences, Le pont de Claix, France	Cat# 559925; RRID: AB_2869266
Streptactin HRP	IBA GmbH, Illkirch, France	2-1502-001
Streptavidin Brillant Violet 650	BioLegend, London, UK	405231

**Critical commercial assays**		

QuickChange II site-directed mutagenesis kit	Agilent Technologies, Les Ulis, France	200523-5

**Deposited data**		

Raw & clinical data	Mendeley Dataset	https://doi.org/10.17632/69tvkst9ct.1

**Experimental models: Cell lines**		

HEK293T cell line	ATCC	CRL-3216

**Oligonucleotides**		

Primers for Spike D614G mutation forward 5’-CTTTATCAGGgCGTGAATTGTAC-3’ reverse 5’-AACTGCAACCTGATTACTG-3’	IDT Leuven, Belgium	this paper

**Software and algorithms**		

Amber10:EHT force field	The Amber project	http://ambermd.org/AmberModels.php
Molecular Operating Environment (MOE) 2018.01 software	Chemical Computing Group Inc	www.chemcomp.com/Products.htm
Prism 7.0 software	GraphPad Software	www.graphpad.com/scientific-software/prism/

### Resource availability

#### Lead contact

Further information and requests for resources and reagents should be directed to and will be fulfilled by the lead contact, Tony Avril (t.avril@rennes.unicancer.fr).

#### Materials availability

This study did not generate new unique reagents.

***Antibodies, plasmids and other reagents*–**All antibodies except those specified below were purchased from Jackson Immunoresearch (Ozyme, Saint-Cyr-L’École, France). We also used the rabbit monoclonal anti-SARS-CoV-2 Spike S1 (Sino Biologicals, Clinisciences, Nanterre, France) antibody. The following secondary antibodies were used: Alexa Fluor (AF) 488 conjugated donkey anti-rabbit IgG, AF488 conjugated F(ab’)2 donkey anti-human IgG, AF647 F(ab’)2 donkey anti-human IgM, FITC conjugated F(ab’)2 goat anti-human IgA (Thermo Fisher Scientific, Illkirch, France), Brilliant Violet 650 conjugated streptavidin (BioLegend, Ozyme), horseradish peroxidase (HRP) conjugated polyclonal goat anti-rabbit IgG (Dako, Agilent, Les Ulis, France), HRP conjugated StrepTactin (IBA GmbH, Fisher Scientific, Illkirch, France), and HRP-conjugated anti-FLAG (Sigma-Aldrich (St Quentin Fallavier, France)). Plasmids pTwist EF1alpha nCoV-2019 S 2xStrep, pLVX EF1alpha nCoV2019 E IRES-Puro and pLVX EF1alpha nCoV2019 M IRES-Puro encoding for SARS-CoV-2 Spike (D614 variant), E and M proteins respectively were a kind gift from the Krogan laboratory (UCSF, San Francisco, CA, USA) (Gordon et al., 2020); and pCMV3 nCoV2019 Spike (D614 variant) C-FLAG Hygro was obtained from Addgene (Teddington, UK) (hereafter named Sf). Plasmid encoding for Spike G614 variant was generated using the pTwist EF1alpha nCoV-2019 S 2xStrep plasmid and the Q5 site-directed mutagenesis kit (New England BioLabs, Evry, France) following the manufacturer’s recommendations. D614 (codon GAC at position 1849) was replaced by G614 (codon GGC at the same position) with the following primers (IDT, Leuven, Belgium): forward 5’-CTTTATCAGGgCGTGAATTGTAC-3’ and reverse 5’-AACTGCAACCTGATTACTG-3’. The sequence of the modified plasmid was further verified after complete sequencing (Integragen, Evry, France). Other reagents not specified below were purchased from Sigma-Aldrich.

***Human sera collection*–** The study was carried out according to the regulation of Rennes Biobank (BRIF number: BB-0033-00056) certified as meeting the requirements of NF S96900 for receipt preparation preservation and provision of biological resources. Serum samples were gathered in the SEROCOV collection (DC-2019-3585). Socio-demographic information, underlying medical conditions, history of symptoms back to January 2020, and history of COVID-19 diagnosis before this investigation were collected at the time of the blood test and were presented in Tables 1 and S1 (entitled ‘raw & clinical data’). Table S1 is available from Mendeley Data at https://doi.org/10.17632/69tvkst9ct.1. Each COVID-19 participant was documented by a positive SARS-CoV-2 RT-PCR on respiratory samples. COVID-19 patients were categorized according to their symptom’s status based on their clinical conditions and care requirement. Patients with symptoms (fever, cough, anosmia, dysgeusia, …) and who did not require hospitalization were classified as mild COVID-19. Patients with symptoms and requiring hospitalization for oxygen therapy were classified as moderate COVID-19. Main patients of this group were cared for in pneumology, emergency (ENT), polyvalent internal medicine, and geriatric units. Severe forms of COVID-19 were defined by patients requiring intensive care unit (ICU), hospitalization, and oxygen therapy (oxygen flow superior to 6L/min or intubated). Patients with hyper-immunoglobulin M syndromes presented lupus pathology with cryoglobulinemia or primary parvovirus B19/EBV infection. Five sera were selected from infected patients with classical seasonal coronaviruses including 3/5 hCoV-OC43, 1/5 hCoV-NL63 and 1/5 hCoV-229E. Pre-pandemic sera were collected residual samples drawn before January 2020; and SARS-CoV-2 infected patients heparinized plasma were obtained from hospitalized patients at Rennes University Hospital Pontchaillou and the Centre Eugène Marquis (Rennes, France) between March 11^th^ and September 15^th^, 2020. All sera were aliquoted and conserved at 4°C for short- term use or frozen at −80°C.

***Cell culture and transfection –*** Human epithelial HEK293T (HEK) cells were grown in Dulbecco's modified Eagle's medium (Gibco, Thermo Fisher Scientific) supplemented with 10% heat-inactivated fetal bovine serum (FBS) in a 5% CO2 humidified atmosphere at 37°C. For transient overexpression of SARS-CoV-2 transmembrane proteins, HEK cells (10^6^) were seeded in a 10 cm Petri dish with 10 mL complete medium for 24 h, and then transfected using calcium phosphate co-precipitation with DNA for 48 h. Plasmids (10 μg per dish) were initially diluted with 0.5 mL of CaCl2 (120 mM) and 0.5 mL of HEPES Buffer Saline solution (2x: HEPES 55 mM, NaCl 274 mM, Na2HPO4 1.4 mM, pH 7.05).

***Western blotting –*** SARS-CoV-2 S, E and M expressing HEK cells were resuspended in ice-cold lysis buffer (composed of 20 mM Tris-HCl, pH 7.5, 150 mM NaCl, 1% Triton X-100) supplemented with protease and phosphatase inhibitor cocktails (Roche, Sigma-Aldrich). Proteins were resolved by SDS-polyacrylamide gel electrophoresis (12% and 7% polyacrylamide gels for viral E and M proteins, and S proteins respectively) and transferred to nitrocellulose membrane for blotting. The membranes were blocked with 3% BSA in 0.1% Tween 20 PBS and incubated with rabbit anti-Spike antibody (1 in 1000 dilution) for Spike (D614 and G614 variants) detection; with HRP-conjugated StrepTactin (1 in 10,000 dilution) for S, E and M detection; or with anti-FLAG (1 in 10,000 dilution) for FLAG-tagged S protein. Anti-Spike antibody binding was detected using HRP-conjugated anti-rabbit secondary antibodies (1 in 7000 dilution) (Dako) and visualized using ECL (KPL, Eurobio, Courtaboeuf, France) according to the manufacturer's instructions. Images were obtained using a G:box imager (Syngene, Fisher Scientific).

***Flow cytometry –*** HEK cells expressing SARS-CoV-2 S, E and M proteins were resuspended using trypsin (Thermo Fisher Scientific) (1 in 5 dilution in PBS). Cells (2.5x10^5^ per well) were distributed in 96-well plates. For analyzing total expression of viral proteins, cells were fixed and permeabilized following the manufacturer's instructions (eBiosciences, Thermo Fisher Scientific). HEK cells were then stained with BV650 conjugated streptavidin (1 in 250 dilution) for 30 min at 4°C. After washes with a permeabilization buffer, cells were resuspended in PBS 2% FBS and directly analyzed by flow cytometry. For Spike surface expression, cells were incubated with rabbit anti-Spike antibody for 30 min at 4°C, washed three times in PBS 2% FBS, and incubated with AF488 conjugated anti-rabbit antibody for 30 min at 4°C. After washes, cells were resuspended in PBS 2% FBS and directly analyzed by flow cytometry. For the serological assay, intact cells were first incubated with sera (1 in 50 dilution in PBS 2% FBS and 5% donkey serum (PBS FBS/DS)) from healthy donors and SARS-CoV-2 infected patients for 30 min at 4°C. Cells were washed in PBS FBS/DS and incubated with AF488 and AF647 conjugated donkey anti-human IgG and IgM F(ab’)2 antibodies or AF488 conjugated goat anti-human IgA F(ab’)2 antibodies for 30 min at 4°C. After washing, the cells were resuspended in PBS FBC/DS containing 7AAD reagent (BD Biosciences, Allschwil, Switzerland) to exclude the dead cell population and directly analyzed using flow cytometry on a Novocyte flow cytometer (Acea Biosciences, Agilent). The population of interest was gated according to its FSC/SSC criteria. The dead cell population was excluded using 7AAD staining. Data were analyzed with the NovoExpress software (Acea Biosciences). For protein expression levels, results were expressed as specific fluorescence intensity given by the ratio of the mean of test / the mean of control (*i.e.* secondary antibodies alone). For Ig binding level, results were expressed as specific Ig binding given by the ratio of specific fluorescence intensity obtained with HEK cells expressing viral transmembrane proteins / specific fluorescence intensity obtained with HEK cells only exposed to the transfection reagent (without DNA).

***Molecular modeling*** - Sequences used for predicted protein structures of Spike D614 variant (PDB ID 6ZB5, EM 2.85Å resolution) and G614 variant (PDB ID 6XS6, EM 3.70Å resolution, lacking the RBD domain) were initially aligned using ClustalOmega. Sequence alignment showed almost a complete identity except for residue D/G614, an RRA insertion at position 681 in 6BZ5, and a PP→KV mutation at residue 983–984 in 6BZ5. In addition, the initial structural analysis of the G614 variant (6SX6) revealed a clear lack of resolved structures, including the loop between T824 and K851. The homology model (HM) using 6SX6 sequence hence yielded an erroneous geometry. Instead, the structure based on the 6BZ5 sequence with a manually introduced D614G mutation was used. All modeling performed using the Molecular Operating Environment (MOE) 2018.01 software (Chemical Computing Group Inc, Montréal, Canada) and Amber10:EHT force field.

***Statistical analyses*** - Graphs and statistical analyses were performed using GraphPad Prism 7.0 software (GraphPad Software). Data are presented as mean ± SD or SEM of at least three independent experiments. Statistical significance (p < 0.05 or less) was determined using a paired or unpaired t test or ANOVA when appropriate.

## Data Availability

Data reported in this paper will be shared by the lead contact upon request. This paper does not report original code.

## Supplemental information

Document S1. Figures S1 and S2

Table S1. Clinical information and raw data obtained from healthy donors and COVID-19 patients, related to Table 1
