# Supplementary material for: SARS-CoV-2 integral membrane proteins shape the serological responses of patients with COVID-19
Source: iScience. 2021 Sep 29;24(10):103185. doi: 10.1016/j.isci.2021.103185 (PMC8479324; doi:10.1016/j.isci.2021.103185)

iScience, Volume *24*

Supplemental information

SARS-CoV-2 integral membrane proteins shape the serological responses of patients with COVID-19

Sophie Martin, Christopher Heslan, Gwénaële Jégou, Leif A. Eriksson, Matthieu Le Gallo, Vincent Thibault, Eric Chevet, Florence Godey, and Tony Avril

Supplemental figure titles and legends

- Figure S1: Serological profile of SARS-CoV-2 infected patients against viral integral membrane proteins according to the disease severity.
- Figure S2: Impact of Spike G614 variant on the seropositivity of SARS-CoV-2 infected patients.
- Table S1: Clinical information and raw data obtained from healthy donors and COVID-19 patients.

Supplemental Figures legends

Figure S1 (related to Table 1, S1 and Figure 2): *Serological profile of SARS-CoV-2 infected patients against viral integral membrane proteins according to the disease severity.* **(A)** Times from blood taken and PCR test and symptoms were represented for the different groups of COVID-19 patients used in this study. **(B)** Results obtained with our serological assay was compared to results obtained with serological assays used for diagnosis from Beckman and Roche companies. **(C)** Sera from control donors and SARS-CoV-2 infected patients were tested for their positivity against viral E proteins using the SARS-CoV-2 serological assay described in Figure 1. Control sera were obtained from heathy donors and collected before January 2020 (CTR, n=38); and from patients infected with others coronavirus (n=5) or patients with hyperimmunoglobulin M syndrome (n=5) (included in CTR2, n=10). Sera collected after January 2020 were obtained from donors without symptoms (no symptom, n=26); with symptoms related to SARS-CoV-2 infection (symptoms, n=4); and from patients positive for SARS-CoV-2 infection (COVID+) and developing mild (blue, n=22), moderate (green, n=14) and severe (orange, n=15) forms of COVID-19. Specific binding of IgG (circles), IgM (squares) and IgA (diamonds) were represented and thresholds (grey boxes) were obtained with the basal levels of Ig binding from control sera. Statistical analysis: unpaired two-tailed *t-test* with Welch's correction comparing CTR versus CTR2 donors and (CTR+CTR2) versus no symptom, symptoms, mild, moderate or severe donors.

Figure S2 (related to Figure 3): *Impact of Spike G614 variant on the seropositivity of SARS-CoV-2 infected patients.* **(A)** Predictive residue interactions with electrostatic, hydrophobic (left panels) and lipophobic (right panels) properties were compared between Spike D614 and G614 variants. **(B-D)** Expression of viral transmembrane Spike D614 and G614 proteins at the surface of HEK cells. HEK cells transfected with viral genes encoding Spike D614 and G614 transmembrane proteins tagged with two Strep Tag II motifs were analyzed for viral protein expression by western-blot **(B)** and flow cytometry **(C-D)** using StrepTactin, streptavidin or anti-Spike antibody. The percentage of positive cells and protein expression levels were represented in **(C)** and **(D)**. Statistical analysis: paired two-tailed *t-test* comparing D614 versus G614 conditions. **(E)** Results obtained with our serological assay with Spike G614 variant was compared to results obtained with serological assays used for diagnosis from Beckman and Roche companies.

FIGURE S1 (related to Table 1, S1 and Figure 2): Serological profile of SARS-CoV-2 infected patients against viral integral membrane proteins according to the disease severity.

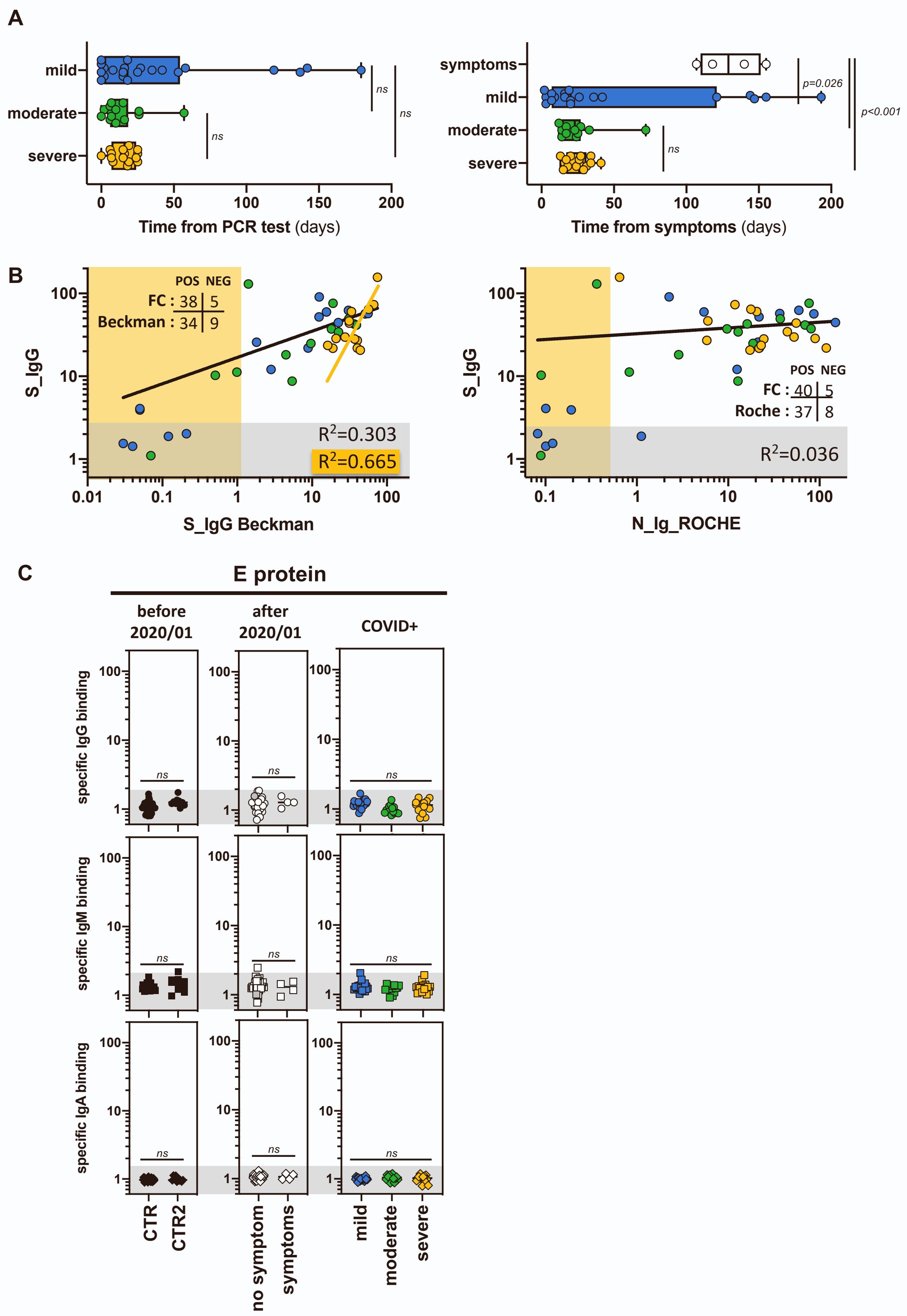

FIGURE S2 (related to Figure 3): Impact of Spike G614 variant on the seropositivity of SARS-CoV-2 infected patients.

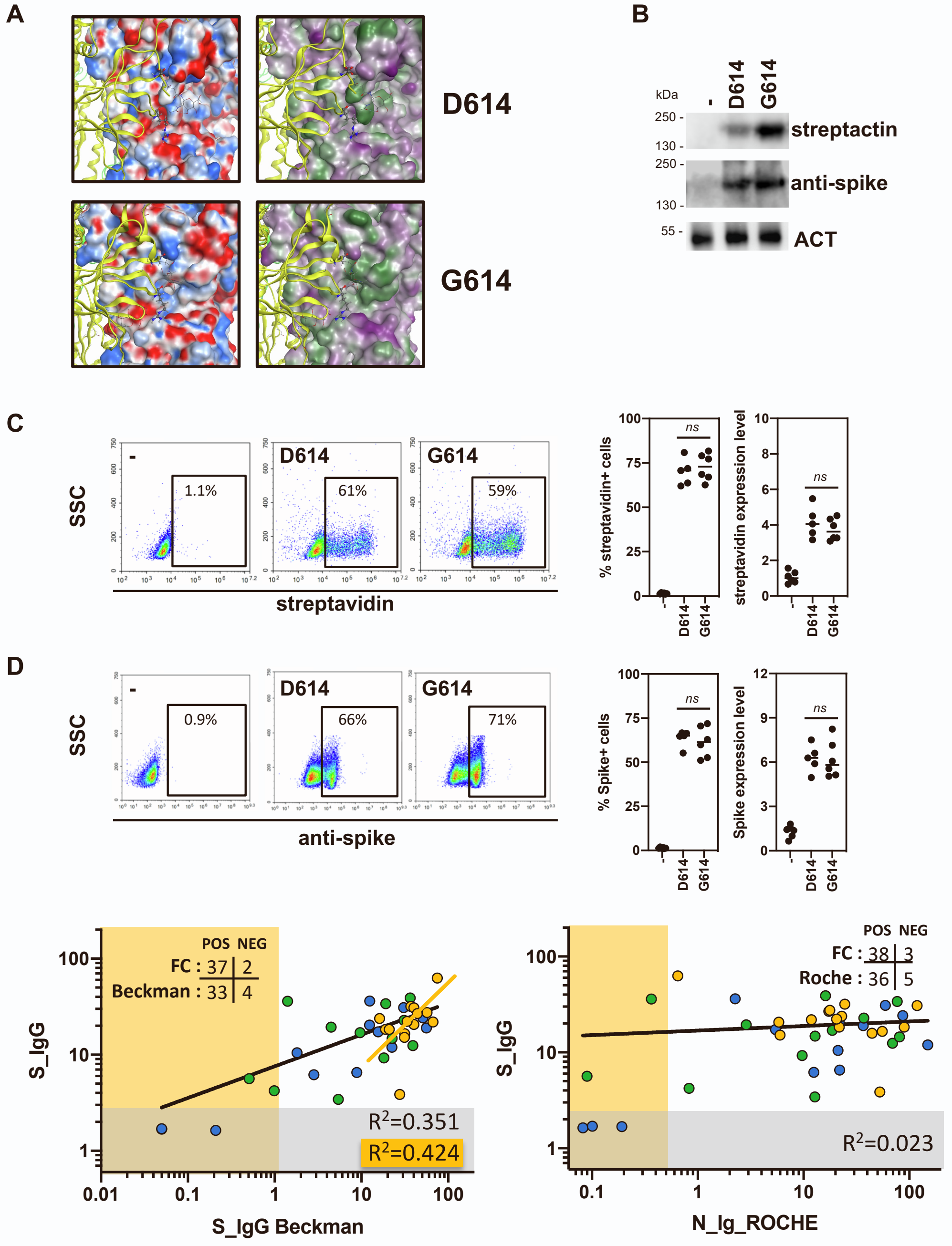

Supplement: Document S1. Figures S1 and S2 [file mmc1.pdf]
